# Minimal Invasive Management of Anastomosis Leakage after Colon Resection

**DOI:** 10.1155/2015/374072

**Published:** 2015-03-16

**Authors:** Esin Kabul Gürbulak, İsmail Ethem Akgün, Ayhan Öz, Sinan Ömeroğlu, Muharrem Battal, Fevzi Celayir, Mehmet Mihmanlı

**Affiliations:** Department of General Surgery, Şişli Hamidiye Etfal Training and Research Hospital, 34371 Istanbul, Turkey

## Abstract

The gold standard of surgical treatment of colorectal anastomotic leak is abdominal drainage of collected fluid and stoma formation. Conventional laparotomy has been the preferred approach for treatment. However, both laparoscopic surgical techniquesand endoscopic stenting have gained popularity over the past years as minimal invasive approaches, especially in the management and treatment of perforations of the gastrointestinal system. We present here a successful treatment with a minimal invasive management of anastomosis leak in the early postoperative period after colon resection in a 62-year-old female patient who had undergone urgent laparoscopic intra-abdominal lavage and drainage followed by endoscopic stenting.

## 1. Introduction

Over the past decade, there has been a soaring interest in endoscopic stenting, especially with the introduction of more sophisticated stents and various stent delivery systems. These new armamentaria have served as alternatives for palliation of symptoms caused by noncurable gastrointestinal malignancy or acute malignant colonic obstructions. Furthermore, they also function as a bridge to surgery thus allowing a single-stage surgical procedure to be performed later [1]. Similarly, their use has been popularized for several benign pathologies including gastrointestinal strictures, fistulas, and perforations [2]. To the best of our knowledge, reports on the role of self-expandable metallic stents (SEMS) in the treatment of colorectal anastomosis leakages are scant in the literature. To date, only one case describing their use, without necessitating stoma formation, has been reported [3]. We present here a successful treatment with a minimal invasive management of anastomosis leak in the early postoperative period after colon resection in a patient who had undergone urgent laparoscopic intra-abdominal lavage and drainage followed by endoscopic stenting.

## 2. Case Presentation

A 62-year-old female patient underwent a laparoscopic anterior resection procedure for sigmoid colon cancer. The patient who was discharged on postoperative day four uneventfully presented with complaints of abdominal pain, distended abdomen, nausea, and fever lasting for 24 hours on 4th day after discharge. Her past medical and surgical history was unremarkable except for a previous diagnosis of systemic lupus erythematosus for which she received prednisolone 16 mg/day as treatment.

On physical examination, she had fever at 38.7 C, blood pressure was 110/60 mm/Hg, heart rate was 100/min, and the respiratory rate was 18/min. She was in mild/acute distress and she was alert and oriented. Abdominal examination was notable for distention, hypoactive bowel sounds, and tenderness in all quadrants. There was guarding and rebound in the lower quadrants of the abdomen.

**Figure 1:**
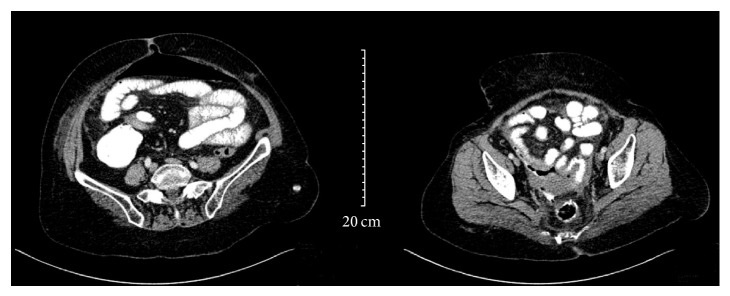
CT of the abdomen, showing extravasation of luminal contrast from the rectosigmoid region into a collection in the pelvis and air bubbles around the anastomosis. There is associated free air in the peritoneum.

Laboratory evaluation was remarkable for leukocytosis 18,000/mm^3^ (4500–10,000/mm^3^), and highly elevated C-reactive protein (CRP) levels of 372 mg/dL (normal range < 5 mg/dL). All other laboratory parameters including electrolytes and renal and liver function tests were within the normal range. A computed tomography (CT) of the abdomen revealed extravasation of luminal contrast from the anastomosis level into the pelvic region (Figure 1). In addition, a collection of fluid measuring 8 × 5 cm was noticed in the pelvis. There was associated free air in the peritoneum.

A diagnosis of peritonitis due to anastomosis leakage was made and urgent surgical intervention was recommended. The laparoscopic approach was planned. The abdomen was entered laparoscopically via the previous trocar sites. On laparoscopic exploration, the anastomosis site was characterized with small bowel adhesions covered with layers of fibrin clots and purulent fluid accumulation in the pelvic region. Aspiration of the purulent fluid was performed followed by separation of the small bowel adhesions. A 1 cm colon peritoneal fistula covered with fibrin layers was identified at the anastomosis site, which localized intraperitoneally. There was no fecal contamination. The abdomen was irrigated with isotonic saline solution and a complete decontamination of the pelvic contents was performed. After adequate drainage of pelvic collection, a drain was placed through the port site at the left lower quadrant across the anastomosis site into the pelvic region and the procedure was ended. The need for an ostomy was precluded as an endoscopic stent was planned for the closure of the anastomosis site. However, because our endoscopy unit had not been work at night time as emergency services, the patient was sent to the endoscopy unit on 5th hour after the operation.

**Figure 2:**
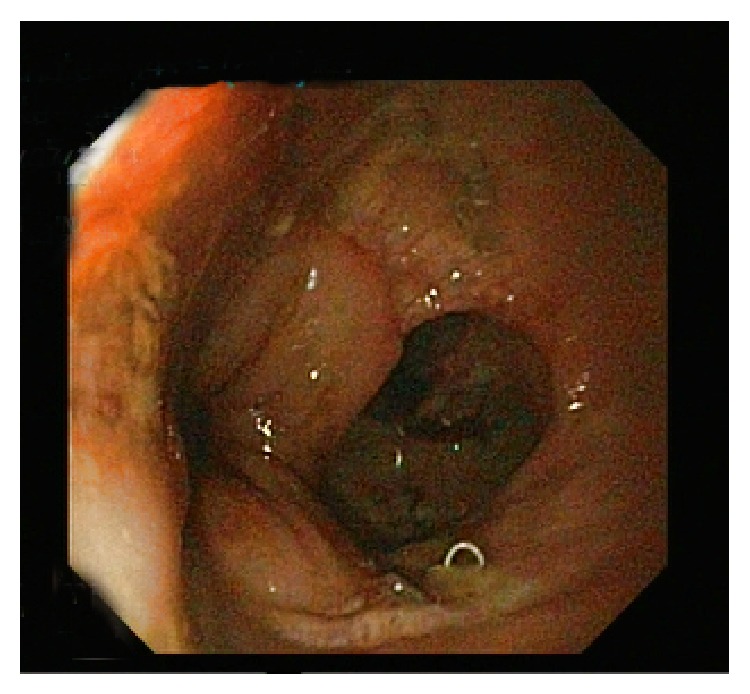
A 1 cm fistula at the site of anastomosis was shown at the sigmoidoscopy.

**Figure 3:**
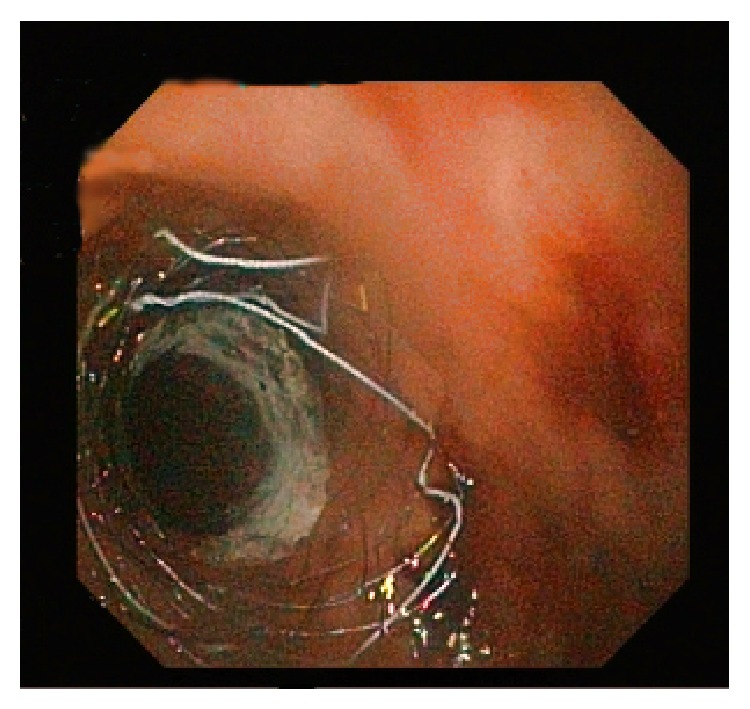
Closure of the fistula with endoscopic stent placement.

**Figure 4:**
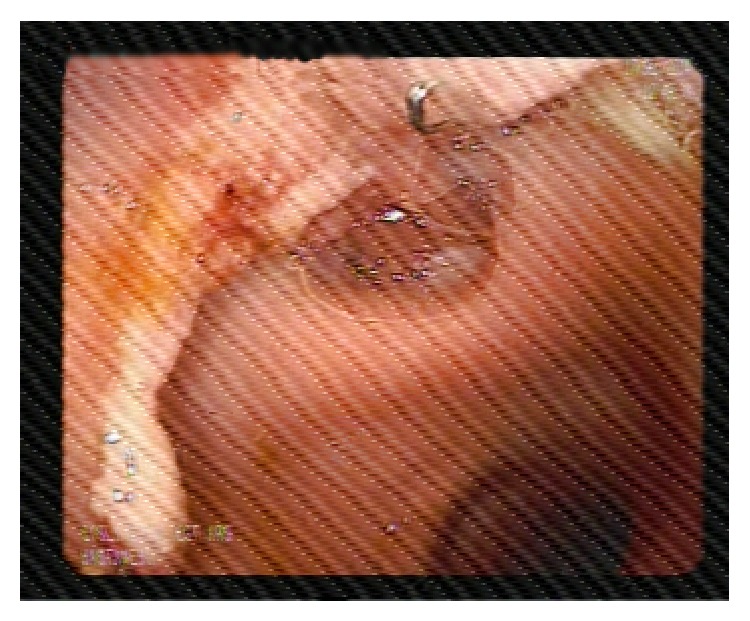
Sigmoidoscopy on 24th day postoperatively, revealing healed anastomotic line and no fistula.

At sigmoidoscopy, the anastomosis site with 1 cm fistula was identified at 17 cm above the anal verge (Figure 2). To close the fistula, a 12 cm long partially coated SEMS (Hanarostent Duodenum/Pylorus Lasso, NCN, MI Tech Co., Seoul, South Korea) was placed across the site of leak successfully (Figure 3). The patient was placed on prophylactic treatment with a third generation cephalosporin and intravenous metronidazole. A dramatic decrease in the patients fever and leukocytosis was observed 24 hours after the procedure. Abdominal pain gradually resolved and oral feeding was started. Abdominal drainage which started at a rate of 150–200 cc/day ceased after 5 days. Follow-up CT demonstrated complete resolution of the anastomosis leak and abscess. The drain was removed and stent removal was planned at four weeks after the procedures. On the seventh postoperative day, the patient was discharged uneventfully. Twenty-four days after the procedure, the patient presented again. She reported an incident of the stent falling out and passing in bowel motion. Sigmoidoscopy was performed and it was consistent with a complete healing of the anastomosis site, while the absence of the stent in the previously inserted location was confirmed (Figure 4). During follow-up, the patients remained well with no complaints with defecation.

## 3. Discussion

Postoperative leakage of anastomosis is one of the major complications of colorectal surgery and its mortality has been reported to range between 6% and 39.3% [4]. The gold standard of surgical treatment is abdominal drainage of collected fluid and stoma formation. Conventional laparotomy has been the preferred approach for treatment. Minimal invasive colorectal surgery has gained popularity over the past years. Also, the use of laparoscopic procedures for the treatment of emergency surgical interventions such as perforated appendicitis, gangrenous perforated cholecystitis, and peptic ulcer perforation has been widely appreciated. Despite the mounting interest, only few reports in the medical literature have investigated the laparoscopic approach to the management of complications such as anastomotic leakages associated with previous laparoscopic colorectal surgery [5]. In this case, we were preferred to begin with the laparoscopic approach because the patient had undergone laparoscopic surgery previously, and we intended not to eliminate all the benefits of laparoscopy. The laparoscopy helped us to conserve all the advantages of laparoscopic surgery, while the technique offers a chance for conversion to open surgery when required.

In our case, on laparoscopic exploration, identified factors which included size of fistula (1 cm), small bowel adhesions covered with layers of fibrin clots, and purulent fluid accumulation without fecal contamination led the plan of peritoneal lavage and drain placement followed by stent closure of the anastomotic leak. This precluded the need for stoma formation.

In patients with peritonitis, disease classification according to risk enables a good prediction of prognosis thus affecting operative decision making. One of the scoring systems used to predict prognosis and mortality-risk is the Mannheim Peritonitis Index (MPI). It has been reported to be a reliable and accurate scoring system that evaluates patients based on preoperative findings. The mortality of patients with a score below 21 according to MPI is known to range between 0% and 2.3% [6]. In our case, operating procedure proceeded on the basis of intraoperative findings of which calculated score of MPI was found to be 20.

The role of endoscopic stents in the management and treatment of perforations of the gastrointestinal system has gained wide attention in recent years. Although there are no randomized controlled trials evaluating the utility of endoscopic stents for anastomotic leakages, their success rates have been reported by systematic reviews to be around 80–85% [7]. While several studies have described the use of SEMs for the closure of esophagojejunostomies, there are few studies describing the experience with colorectal anastomosis leakages [7–10].

Endoscopic SEMS placement can serve as an early treatment alternative for closure of small sized colonic anastomosis that are not associated with severe sepsis. In our case, the anastomosis site was 1 cm in size. At an early stage, laparoscopic lavage and drainage of the pelvic fluid collection were performed to prevent progression into sepsis. This was followed by the closure of the fistula on the anastomosis site with an endoscopic stent. To ensure successful outcomes from treatment, an early intervention as employed in our case is necessary to prevent progression to sepsis.

On the other hand, SEMs associated complications can also contribute to unsuccessful treatment results. Among the complications associated with endoscopic stents are stent migration, perforation, and hemorrhage. A higher probability of stent migration can lead to failure in the treatment of fistula in the colonic anastomotic site, thus contributing to unsuccessful endoscopic treatment [10]. Rate of migration is reported to be especially higher in fully covered stents than uncovered or partially covered stents [7]. Accordingly, full covered stents are not good options for the treatment of nonstricture perforations. Conversely, as a result of newly formed granulated tissues, the removal of uncovered or partially covered stents is usually difficult to remove, thus creating a risk for colon perforation and laceration [7, 10]. A partially covered stent was preferred in our case primarily due to anastomosis dehiscence that was observed during the early postoperative period that was a short duration for healing and stricture formation; thus, the risk of stent migration was lower for partially covered stents than fully covered stents. Notwithstanding, the partially covered SEMs fell off by itself on the 24th day; complete healing of the anastomosis site was observed by then.

The latest innovations in endoscopic stent technology have brought up biodegradable stents. The property of not being able to remove them is the most important advantage. Additionally, they provide contribution to patients' life qualities, especially in cases of benign strictures refractory to dilatations in gastrointestinal tract and present as an attractive treatment method.

However, published reports on clinical efficacy are limited to patients with anastomotic colorectal strictures and fistulae [11, 12]. Furthermore, the price of these stent is an important limitation. Secondary to these causes, biodegradable endoscopic stents are not most widely used in our clinic, and because of that there is not enough experience; metallic stents are routinely used as in our case.

In summary, we describe the use of laparoscopic drainage and endoscopic stenting in the early postoperative management of peritonitis that occurred as a result of anastomosis leakage, after colon resection. The traditional management of choice for postoperative peritonitis that occurs as a result of anastomosis leakage after colon resections is drainage via laparotomy and defunctioning stoma formation. In patients with minimal risk for sepsis, as an alternative to traditional laparotomy, early interventions with minimal invasive procedures such as laparoscopy may help in the evaluation of the extent of peritonitis and anastomosis leakage. Similar to our case, smaller leakage sites may benefit from colonic stenting for closure after laparoscopic peritoneal lavage and drainage, thus excluding the need for stoma formation. This may help in the reduction of surgical risk for a selected group of patients considered appropriate for minimal invasive surgery. However, in order to establish the safety and efficacy of this technique similar prospective studies involving a larger cohort of patients are required.
